# Population pharmacokinetics of intravenous fosfomycin: dose optimization for critically ill patients with and without kidney replacement therapy

**DOI:** 10.1128/aac.01779-24

**Published:** 2025-05-05

**Authors:** Katharina M. Götz, Sascha Kreuer, Anke-Katrin Volz, Suzanne L. Parker, Jason A. Roberts, George Dimopoulos, Thomas Dimski, Detlef Kindgen-Milles, Lisa K.V. Beuche, Jan T. Kielstein, Thorsten Lehr

**Affiliations:** 1 Saarmetrics GmbH, Saarland University 9379, Saarbrücken, Germany; 2 Department of Clinical Pharmacy, Saarland University 9379, Saarbrücken, Germany; 3 Department of Anaesthesiology, Intensive Care and Pain Therapy, Saarland University Medical Center and Saarland University Faculty of Medicine 197751, Homburg, Germany; 4 University of Queensland Centre for Clinical Research, Faculty of Medicine,The University of Queensland, Brisbane, Queensland, Australia; 5 Herston Infectious Diseases Institute (HeIDI), Metro North Health, Brisbane, Australia; 6 Departments of Pharmacy and Intensive Care Medicine, Royal Brisbane and Women’s Hospital, Brisbane, Australia; 7 UR UM 103, University of Montpellier, Division of Anesthesia Critical Care and Emergency and Pain Medicine, Nimes University Hospital, Nimes, France; 8 Third Department of Critical Care Medicine, National and Kapodistrian University of Athens, Medical School 68993, Athens, Greece; 9 Department of Anesthesiology, University Hospital Düsseldorf, Heinrich Heine University Düsseldorf, Düsseldorf, Germany; 10 Medical Clinic V (Nephrology, Rheumatology, Blood Purification), Academic Teaching Hospital Braunschweig, Braunschweig, Germany; Providence Portland Medical Center, Portland, Oregon, USA

**Keywords:** fosfomycin, population pharmacokinetics, kidney replacement therapy, dose optimization, critically ill

## Abstract

We investigated the pharmacokinetics (PK) of intravenous fosfomycin in critically ill patients undergoing different types of kidney replacement therapy (KRT) to identify optimized dosing regimens for patient care. Four prospective, observational studies contributed data of critically ill patients with prolonged-intermittent KRT (PIKRT, *n*=18), continuous KRT (CKRT, *n*=15), or without KRT (*n*=12) for population PK analysis. Subsequently, licensed daily dosages (12–24 g), varying estimated glomerular filtration rates (eGFR, 0–120 mL/min/1.73 m^2^), and scenarios with or without KRT were simulated for comparison against minimum inhibitory concentrations (MICs, 32–256 mg/L). A dosing regimen was considered “effective” if the ratio of area under the concentration–time curve from 24–48 hours and MIC (AUC_24-48_/MIC ratio) exceeded 22.7 or 83.3, respectively, and if the percentage of time above MIC between 24 and 48 hours (%T_24-48_>MIC) was greater than 69.0. The probability of target attainment (PTA) was assessed using Monte Carlo simulations (*n* = 2,000) for each scenario. A two-compartment model incorporating body (1.6 L/h) and dialysis (2.0 L/h) clearance identified eGFR, dialyzate flow rate (Q_D_), and time after first dose as significant covariates. Considering the AUC_24-48_/MIC ratio, 8 g three times daily (TID) was bactericidal (PTA≥90%) in all scenarios at MIC_32_, 5 g TID was bacteriostatic (PTA≥90%) at MIC_64_, and 8 g TID was also effective (PTA≥90%) at MIC_128_. Based on the %T_24-48_>MIC, 4 g TID and 8 g TID were bactericidal (PTA≥90%) at MIC_32_ and MIC_64_, respectively. In conclusion, a dosage of 12–24 g/d intravenous fosfomycin is plausible for critically ill patients undergoing CKRT or PIKRT.

## INTRODUCTION

Critical illness can significantly alter drug exposure (1) due to a variety of factors, including insufficient renal clearance and variations in the volume of distribution. For critically ill patients with acute kidney injury (AKI) or those with a preexisting need for dialysis, kidney replacement therapy (KRT) further affects the elimination of drugs and challenges the selection of effective antibiotic doses from dosing recommendations established in the absence of KRT (2, 3).

Fosfomycin, a bactericidal broad-spectrum antibiotic, is valuable for treating critically ill patients (4–6) due to its favorable susceptibility profile and extensive tissue penetration (7–10). It is a small, hydrophilic molecule with negligible protein binding in serum and primary elimination by the kidneys (11). Fosfomycin is also not metabolized. These characteristics make it highly dialyzable and, thus, a potential treatment option for critically ill patients with KRT. Intravenous (IV) fosfomycin is approved for use in daily doses of 12–24 g, divided into two or three administrations, adjusted based on the indication and kidney function (12, 13). Overall, IV fosfomycin is well-tolerated (14–16).

Regulatory guidelines require knowledge on the pharmacokinetics (PK) of drugs that are predominantly eliminated by the kidneys (17, 18), and population PK modeling is a useful tool to discriminate the effects of kidney function and KRT on drug clearance, facilitating dose optimizations for specific patient populations (19, 20). Our previous research indicated that IV doses of 5 g three times daily (TID) are required to achieve appropriate fosfomycin concentrations for patients with urinary creatinine clearance ≥50 mL/min receiving continuous veno-venous hemodialysis (CVVHD) (21). However, the response to fosfomycin may vary with other KRT modalities because continuous KRT (CKRT) uses considerably lower flow rates than, for instance, prolonged-intermittent KRT (PIKRT). Previous studies reported fosfomycin dosing recommendations for patients undergoing PIKRT (22–24), which characterized the PK of fosfomycin using noncompartmental analyses without adjusting for covariates and calculated the dialysis clearance using differences between pre- and post-filter concentrations and blood flow rates (Q_B_), which restrains the generalizability of dosing recommendations to different study or KRT characteristics.

Therefore, the aims of this analysis were (i) to combine the heterogeneous data sets of four fosfomycin PK studies in critically ill patients (21, 23–25), to (ii) develop a comprehensive PK model of IV fosfomycin accounting for KRT (21, 23–25), and (iii) to establish optimized fosfomycin dosing regimens for various critically ill patient scenarios, including the use of CKRT or PIKRT.

## MATERIALS AND METHODS

### Patients and study design

**Fig 1:**
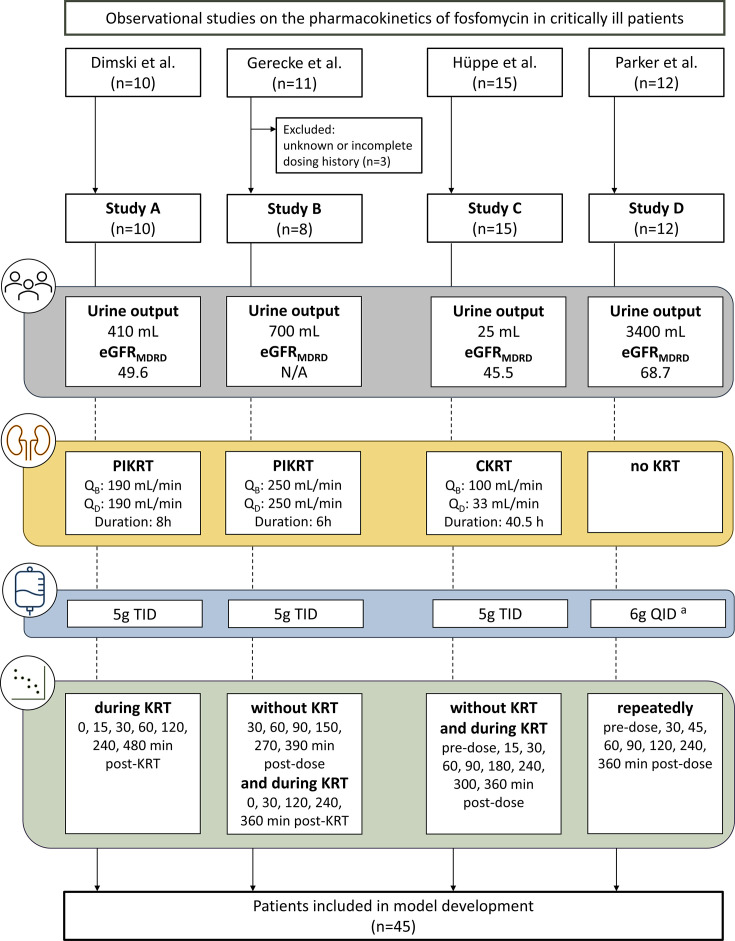
Study characteristics. Values presented as median. Urine output refers to the 24-hour urine output. CKRT, continuous kidney replacement therapy; eGFR_MDRD_, estimated glomerular filtration rate in mL/min/1.73 m^2^ calculated using the Modification of Diet in Renal Disease equation; PIKRT, prolonged-intermittent kidney replacement therapy; KRT, kidney replacement therapy; Q_B_, blood flow rate; Q_D_, dialyzate flow rate; QID, four times a day; TID, three times a day. ^a^4 g four times daily in one patient and 6 g three times daily in another patient.

This analysis used pooled data from four prospective, observational PK studies in critically ill patients undergoing KRT or not receiving KRT, as illustrated in Fig. 1 (21, 23–25). Following the approval by the relevant local ethics committees, all studies were conducted in accordance with the guidelines of the Declaration of Helsinki. All patients or legal representatives provided written informed consent prior to study participation.

Fosfomycin was administered IV in doses of 5 g TID in patients with KRT and 4 g four times daily (QID) or 6 g TID in patients without KRT. The infusion duration was either 30–60 minutes (studies A–B, D) or 120 minutes (study C). Two studies (A, B) included patients with PIKRT, one study (C) included patients with CKRT, and one study (D) involved patients without KRT. Detailed information on the specific dialyzers is provided in the Supplementary Material. The duration of PIKRT episodes varied according to local guidelines, 8 hours in study A and 6 hours in study B. In study C, the duration of CKRT was individually adjusted by treating physicians.

In study A, fosfomycin serum concentrations were collected at the start of KRT and after 15, 30, 60, 120, 240, and 480 minutes. In study B, fosfomycin plasma concentrations were measured 30, 60, 90, 150, 270, and 390 minutes after the first dose without PIKRT. In addition, blood samples were collected before PIKRT and after 30, 120, 240, and 360 minutes. In study C, fosfomycin plasma concentrations were measured 5 minutes before drug administration and after 15, 30, 60, 90, 180, 240, 300, and 360 minutes, both with and without CKRT. In study D, one to six series of fosfomycin plasma concentrations were collected before drug administration and after 30, 45, 60, 90, 120, 240, and 360 minutes. Fosfomycin concentrations were obtained using validated methods, including HPLC/MS (21, 25) and LC-MS/MS (23, 24).

### Data analysis

The data set comprised fosfomycin concentration measurements, KRT characteristics, and data gathered during routine care, such as patient characteristics and laboratory data. Kidney function was assessed by the measured creatinine clearance using 24-hour urine output (*Cr*CL, mL/min), the estimated creatinine clearance using the Cockcroft–Gault equation (e*Cr*CL_CG_, mL/min) (26), and the estimated glomerular filtration rate (eGFR, mL/min/1.73 m^2^) using equations from the Modification of Diet in Renal Disease (MDRD) study and Chronic Kidney Disease Epidemiology Collaboration (CKD-EPI) (27). Table S1 provides all equations that were used to calculate the kidney function.

As indicated by Kidney Disease Improving Global Outcomes (KDIGO), the eGFR was classified by the following categories: <15, 15–29, 30–44, 45–59, 60–89, and ≥ 90 mL/min/1.73 m^2^ (28). Body surface area (BSA) was calculated using the Mosteller formula: BSA (m^2^) = [(body weight (kg) ×body height (cm)) / 3600]^1/2^. Study B, comprising 17.8% of all patients, did not include information on individual body height and laboratory markers. Furthermore, serum potassium and sodium levels were not available for 44.4% of all patients (studies B and D), whereas 24-hour urine output (*Cr*CL) was missing for 40.0% of all patients (studies A and B). Overall, a median (range) of 4 (0–10) serum creatinine and 3 (1–9) 24-hour urine output (*Cr*CL) measurements were available per patient. We employed median imputation for entirely missing patient characteristics. For continuous laboratory data, missing values were imputed using the individual last recorded values. Due to limited data on serum sodium and serum potassium levels, evaluating potential adverse effects like hypernatremia and hypokalemia was not possible.

Population PK modeling and simulation analyses were performed using the nonlinear mixed-effects modeling technique implemented in the software NONMEM (version 7.4, ICON Development Solutions, Ellicott City, MD, USA). The first-order conditional estimation with interaction was used for parameter estimation. Interindividual variability (IIV) was explored using exponential random-effects models. Several statistical models were tested to establish the residual variability. Model selection was based on the NONMEM objective function value (−2 log likelihood, OFV), and differences in OFV (dOFV) were calculated for statistical comparison. A reduction of 3.84 points in the OFV was considered to prove a statistically significant difference (*P* < 0.05) between the nested models varying in one additional parameter (1 *df*). Furthermore, goodness-of-fit plots and the precision of parameter estimates (relative standard errors [RSE] <30%) were considered for model selection. The final model was evaluated using a prediction-corrected visual predictive check (pcVPC) based on 1000 replicates of the original data set. The simulated fosfomycin concentrations were overlaid with the observed data, and the respective median, 5th, and 95th percentiles were compared. R (version 3.6.3, R Foundation for Statistical Computing, Vienna, Austria) and R Studio (version 1.4.1717, RStudio, Inc., Boston, MA, USA) were used for data set generation, statistical analysis, and creation of graphics.

### Model development

One-, two-, and three-compartment models were evaluated as structural models, with *a priori* implementation of two parallel clearance processes to account for fosfomycin elimination via body clearance (CL_body_) and dialysis clearance (CL_KRT_). CL_body_ was fixed to 0 in patients presenting 24-hour urine output <100 mL. Additionally, CL_KRT_ was fixed to 0 for patients not receiving KRT or time periods between KRT sessions. Subsequently, we examined the effects of the dialyzate flow rate (Q_D_) and Q_B_ on CL_KRT_ by implementation of the Michaels equation (29). In addition, the relationship between KRT characteristics and CL_KRT_ was investigated using linear, power, and maximum effect (E_max_) models. The most appropriate base model was selected, and a covariate analysis was performed to examine the available patient and KRT characteristics for statistically significant effects on fosfomycin PK. First, we examined the relationship between kidney function and CL_body_ using linear, power, and E_max_ models. Here, we tested the 24-hour urine output, e*Cr*CL_CG_, eGFR_MDRD_, and eGFR_CKD-EPI_ as markers for kidney function. Subsequently, we evaluated the effects of KRT (yes/no), numbers of KRT episodes, sex, age, body weight, BSA, time after the first dose, time after dialysis start, e*Cr*CL_CG_, eGFR_MDRD_, eGFR_CKD-EPI_, as well as serum concentrations of creatinine, potassium, and sodium on all model parameters. The covariate candidates were examined in sequential forward inclusion (dOFV = 3.84, *P* = 0.05, *df* = 1) and backward elimination (dOFV = 6.63, *P* = 0.01, *df* = 1) procedures.

### Simulations

The final model was used to perform Monte Carlo simulations (*n* = 2,000), considering different dosing regimens within the licensed daily dose range (4, 5, and 8 g TID, 8 g twice daily [BID], 4 g QID), varying kidney function (eGFR_MDRD_ of 0–120 mL/min/1.73 m^2^), and scenarios with CKRT (Q_D_ = 42 mL/min) for 48 hours or PIKRT (Q_D_ = 250 mL/min) for 8 hours on the second day of treatment, as well as without KRT.

The European Committee on Antimicrobial Susceptibility Testing (EUCAST) proposed epidemiological cut-off (ECOFF) values of 32 and 256 mg/L for *Staphylococcus aureus* and *Pseudomonas aeruginosa*, respectively (30). Hence, to identify appropriate dosing regimens, the median of the simulated fosfomycin concentrations was compared with minimum inhibitory concentrations (MICs) of 32, 64, 128, and 256 mg/L. Additionally, we calculated the ratio of the area under the concentration–time curve between 24 and 48 hours and the MIC (AUC_24-48_/MIC ratio) as the exposure-dependent PK/pharmacodynamic (PK/PD) index and the percentage of time that fosfomycin concentrations exceeded the MIC between 24 and 48 hours (%T_24-48_>MIC) as the time-dependent PK/PD index. The AUC_24-48_/MIC ratio target of 22.7 was used to evaluate the bacteriostatic activity against *Enterobacteriaceae* (31). For the evaluation of effective dosing regimens with the bactericidal activity, targets of AUC_24-48_/MIC ratio = 83.3 and %T_24-48_>MIC = 69.0 were applied, both specific to *Enterobacteriaceae* (31). Monte Carlo simulations (*n* = 2,000) and both PK/PD indices were used to assess the probability of target attainment (PTA) for each simulated scenario at MICs between 4 and 512 mg/L.

## RESULTS

### Patients

Table 1 provides a summary of patient characteristics and laboratory parameters. The final data set comprised 727 fosfomycin concentrations from 45 critically ill patients with AKI or chronic kidney disease (CKD). The median (interquartile range) eGFR_MDRD_ was 48.4 (33.3–77.2) mL/min/1.73 m^2^, whereas ten patients (22.2%) presented with anuria. The patients who participated in studies A–C (73.3%) underwent KRT, involving PIKRT (*n* = 18) and CKRT (*n* = 15). Study D included critically ill patients who did not require any KRT (*n* = 12).

**TABLE 1:** Summary of demographic and clinical patient characteristics^*a*^^,*b*^

Characteristics	Study A^23^ (*n* = 10)	Study B^24^ (*n* = 8)	Study C^21^ (*n* = 15)	Study D^25^ (*n* = 12)	P-value^*c*^	Entire cohort (*n* = 45)
Age, years	75.5 (71–79.2)	64 (61.5–77)	57 (55–62)	62.5 (57.8–75)	0.0202	63 (57–75)
Male, n (%)	9 (90%)	4 (50%)	13 (87%)	8 (67%)	0.138	34 (76%)
Female, n (%)	1 (10%)	4 (50%)	2 (13%)	4 (33%)	0.137	11 (24%)
Body weight, kg	76.5 (72.8–79.5)	99.5 (78.8–107)	89 (75–105)	71.5 (69.5–80)	0.0558	80 (70–90)
Height, cm	176 (173–180)	N/A^*e*^	176 (172–180)	166 (160–178)	0.116	175 (168–180)
Body mass index, kg/m^2^	24.1 (23.7–24.6)	N/A	30.1 (23.5–33.2)	26.4 (23.9–27.6)	0.197	24.8 (23.7–30.1)
BSA, m^2^	1.95 (1.87–1.97)	N/A	2.06 (1.94–2.29)	1.82 (1.76–1.96)	0.028	1.96 (1.83–2.05)
Q_B_, mL/min	190 (28–190)	250 (240–250)	100 (100–100)	N/A	<0.001^*d*^	100 (100–150)
Q_D_, mL/min	190 (28–190)	250 (240–250)	33 (33–42)	N/A	<0.001^*d*^	42 (33–50)
Potassium, mmol/L	4.8 (4.2–4.9)	N/A	4.2 (4–4.5)	N/A	0.013^*d*^	4.3 (4–4.5)
Sodium, mmol/L	140 (140–150)	N/A	140 (140–150)	N/A	0.734^*d*^	140 (140–150)
**Creatinine**						
Serum, mg/dL	1.4 (0.98–1.6)	N/A	1.5 (1–2.2)	0.95 (0.6–1.7)	0.198^*d*^	1.3 (0.94–2.1)
Urine, mg/dL	N/A	N/A	0 (0–8)	24 (5.3–35)	0.534^*d*^	0 (0–30)
24-hour urine output, mL	410 (0–2200)	700 (500–1000)	25 (0–820)	3400 (2600–5800)	<0.001^*d*^	410 (0–1400)
**Kidney function**						
*Cr*CL, mL/min	N/A	N/A	0 (0–11.7)	58 (19.2–100)	0.0711^*d*^	0 (0–31.1)
e*Cr*CL_CG_, mL/min	50.5 (48.5–65.4)	N/A	56.7 (42.5–87.6)	61.1 (46.8–122)	0.29^*d*^	56.3 (45.3–89.5)
eGFR_MDRD_, mL/min/1.73 m^2^	49.6 (43.7–75.6)	N/A	45.5 (30.9–72.3)	68.7 (38.9–119)	0.36^*d*^	48.4 (33.3–77.2)
eGFR_CKD-EPI_, mL/min/1.73 m^2^	49.6 (45–74.8)	N/A	49.1 (32.1–74.3)	72.3 (40.6–102)	0.27^*d*^	51.3 (33.9–80.4)
**eGFR**_**MDRD**_ **category**						
≥ 90 mL/min/1.73 m^2^	1 (1)	N/A	2 (0)	4 (0)		7 (1)
60–89 mL/min/1.73 m^2^	2 (0)	N/A	3 (2)	3 (0)		8 (2)
45–59 mL/min/1.73 m^2^	4 (2)	N/A	1 (1)	1 (0)		6 (3)
30–44 mL/min/1.73 m^2^	3 (0)	N/A	5 (1)	4 (0)		12 (1)
15–29 mL/min/1.73 m^2^	0 (0)	N/A	4 (3)	0 (0)		4 (3)
<15 mL/min/1.73 m^2^	0 (0)	N/A	0 (0)	0 (0)		0 (0)

^*a*^ Values are shown as median (interquartile range), unless specified otherwise. eGFR_MDRD_ categories at first dose administration are presented as number and number of anuric patients (in parenthesis), with anuria defined as 24-hour urine output < 100 mL.

^*b*^ BSA, body surface area calculated using the Mosteller equation; *Cr*CL, measured creatinine clearance calculated using 24-hour urine output; e*Cr*CL_CG_, estimated creatinine clearance calculated using the Cockcroft–Gault equation; eGFR_CKD-EPI_, estimated glomerular filtration rate calculated using Chronic Kidney Disease Epidemiology Collaboration equation; eGFR_MDRD_, estimated glomerular filtration rate calculated using the Modification of Diet in Renal Disease equation; Q_B_, blood flow rate; Q_D_, dialysate flow rate.

^*c*^ The continuous variables were compared using the Kruskal–Wallis test, and differences in frequency between groups were compared using the chi-square test.

^*d*^ For repeated measurements, linear mixed-effects models were fitted using the R package lme4 (version 1.1-35.6) to predict variables with study groups. The models included random intercepts for patients nested within study groups. ANOVA *P*-values were obtained using F-tests based on Satterthwaite’s method.

^*e*^ N/A, not available.

A *post-hoc* Dunn test indicated statistically significant differences between studies A and C for age (*P* < 0.05), as well as studies C and D for BSA (*P* < 0.05). In study A, fosfomycin concentrations were measured during PIKRT, while CKRT was partially applied during the observation period. *Post-hoc* comparisons using least-squares means indicated a statistically significant difference between studies A and C for both Q_B_ (*P* < 0.001) and Q_D_ (*P* < 0.001), as well as potassium (*P* < 0.05). Furthermore, pairwise comparisons using least-squares means indicated statistically significant differences between studies A and C (*P* < 0.001), as well as studies A and D (*P* < 0.001), for urine output.

**Fig 2:**
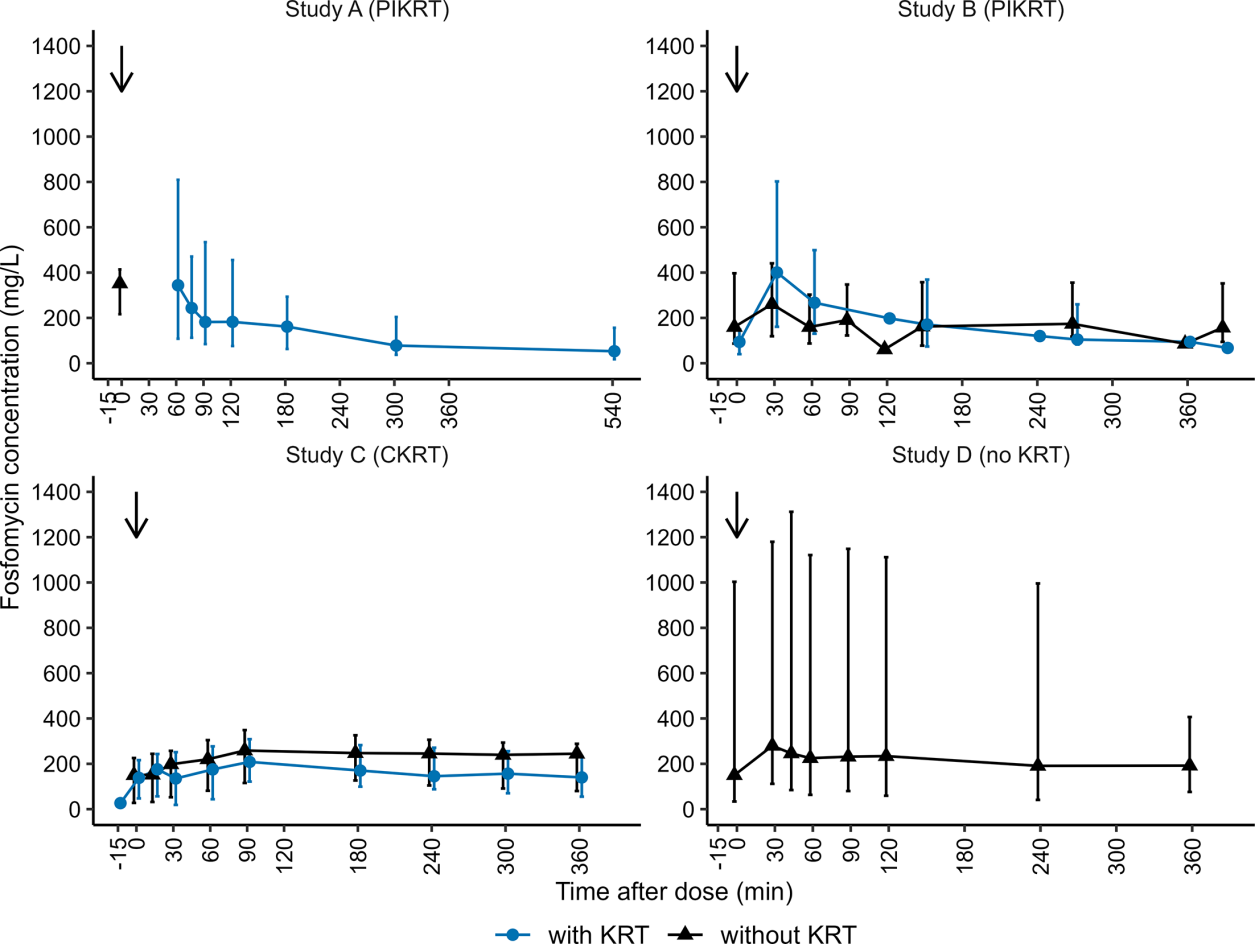
Median fosfomycin concentration–time profiles stratified by study and treatment with KRT. The observed data are shown in blue (with KRT) and black (without KRT). The lines and whiskers represent the respective median, with whiskers indicating the 5th–95th percentile range. The black arrows depict the dosing of fosfomycin. Detailed information about dosing regimens and study populations is given in Fig. 1 and Table 1, respectively. CKRT, continuous kidney replacement therapy; KRT, kidney replacement therapy; PIKRT, prolonged-intermittent kidney replacement therapy.

Figure 2 shows median fosfomycin concentration–time profiles stratified by study and presence of KRT, indicating a high variability between patients. In study D, four patients had eGFR_MDRD_ values ≤45 mL/min/1.73 m^2^ during the observation period, whereas two patients had eGFR_MDRD_ values ≥200 mL/min/1.73 m^2^. This wide range of kidney functions may explain the highly variable fosfomycin exposure observed in study D.

### Population pharmacokinetic model

**Fig 3:**
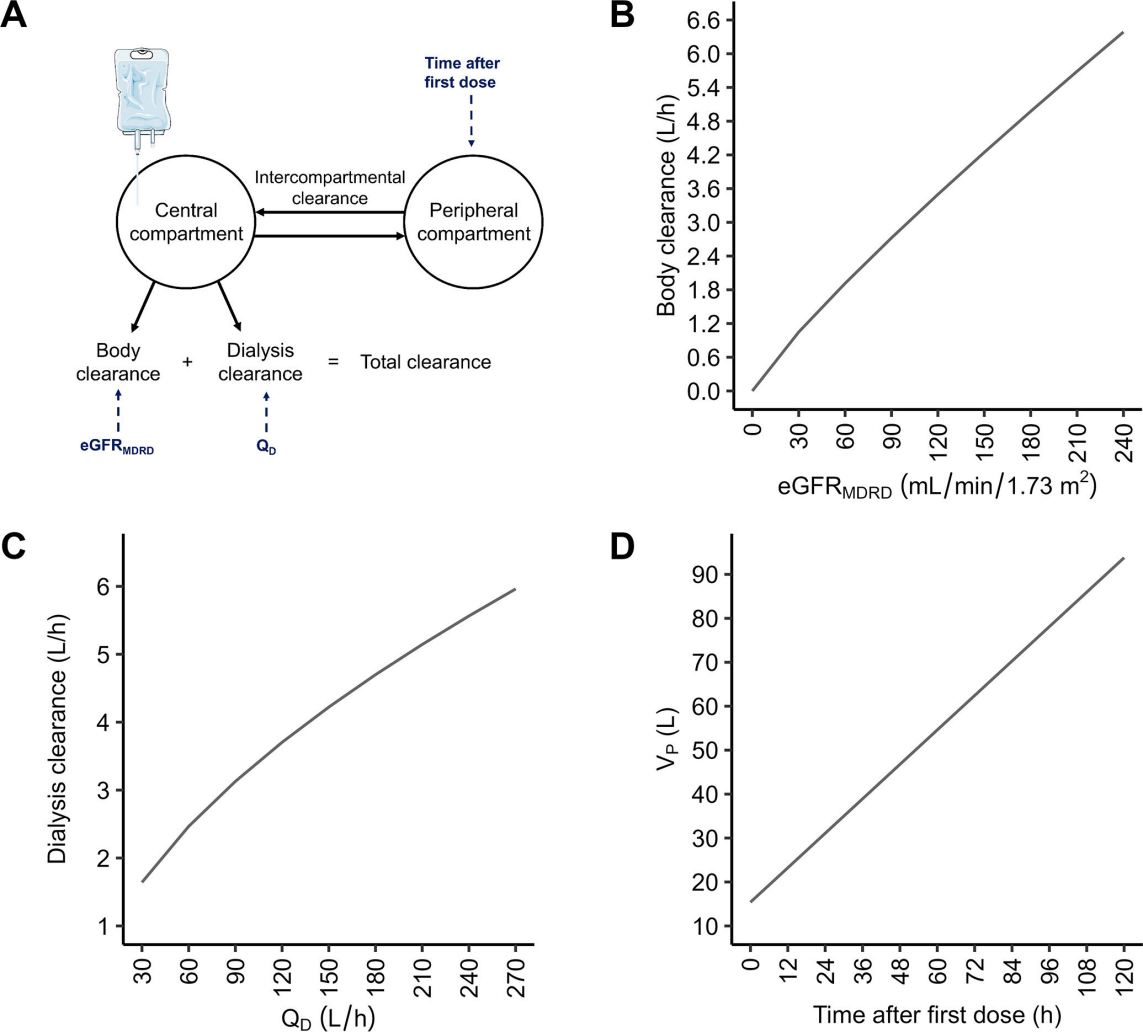
Illustration of the final population pharmacokinetic model and visualized relation of model parameters and covariates. (**A**) Schematic illustration of the final model. (**B–D**) Simulated relationship of the respective model parameter and covariate. eGFR_MDRD_, estimated glomerular filtration rate calculated using Modification of Diet in Renal Disease equation; Q_D_, dialyzate flow rate; V_P_, peripheral volume of distribution.

The concentration–time profiles of IV fosfomycin were best described using a two-compartment model incorporating concomitant body and dialysis clearance (Fig. 3A). The term “body clearance” was used to acknowledge that fosfomycin elimination may involve renal and non-renal components. IIV was supported on CL_body_ and central and peripheral volumes of distribution. CL_body_ was fixed to 0 for study B since these patients presented no fosfomycin elimination without KRT (Fig. 2), which led to individual CL_body_ estimates close to zero. The typical CL_body_ was estimated at 1.6 L/h and associated with large IIV (84.6 %CV). CL_body_ increased with increasing eGFR_MDRD_ (in mL/min/1.73 m^2^) in a nonlinear relationship (Fig. 3B). Subsequently, absolute eGFR_MDRD_ values in mL/min were calculated from the relative eGFR_MDRD_ values in mL/min1.73 m^2^ using the individual BSA. The implementation of absolute eGFR_MDRD_ values did not offer a statistically significant improvement (*P* > 0.05) over relative eGFR_MDRD_ values, although median values of relative and absolute eGFR_MDRD_ presented statistically significant differences (*P* < 0.05, data not shown). CL_KRT_ increased nonlinearly over the Q_D_ range (Fig. 3C). The peripheral volume of distribution (V_P_) increased linearly by 0.07% per minute after the first dose (*P* < 0.001, Fig. 3D), which explained substantial parts of the associated IIV, reducing it from 247.0 %CV to 71.1 %CV. As this variation in the distribution of fosfomycin may primarily occur in patients experiencing fluid retention, resulting from fluid administration during periods of significantly reduced kidney function, the increasing V_P_ was restricted to patients experiencing anuria.

**Fig 4:**
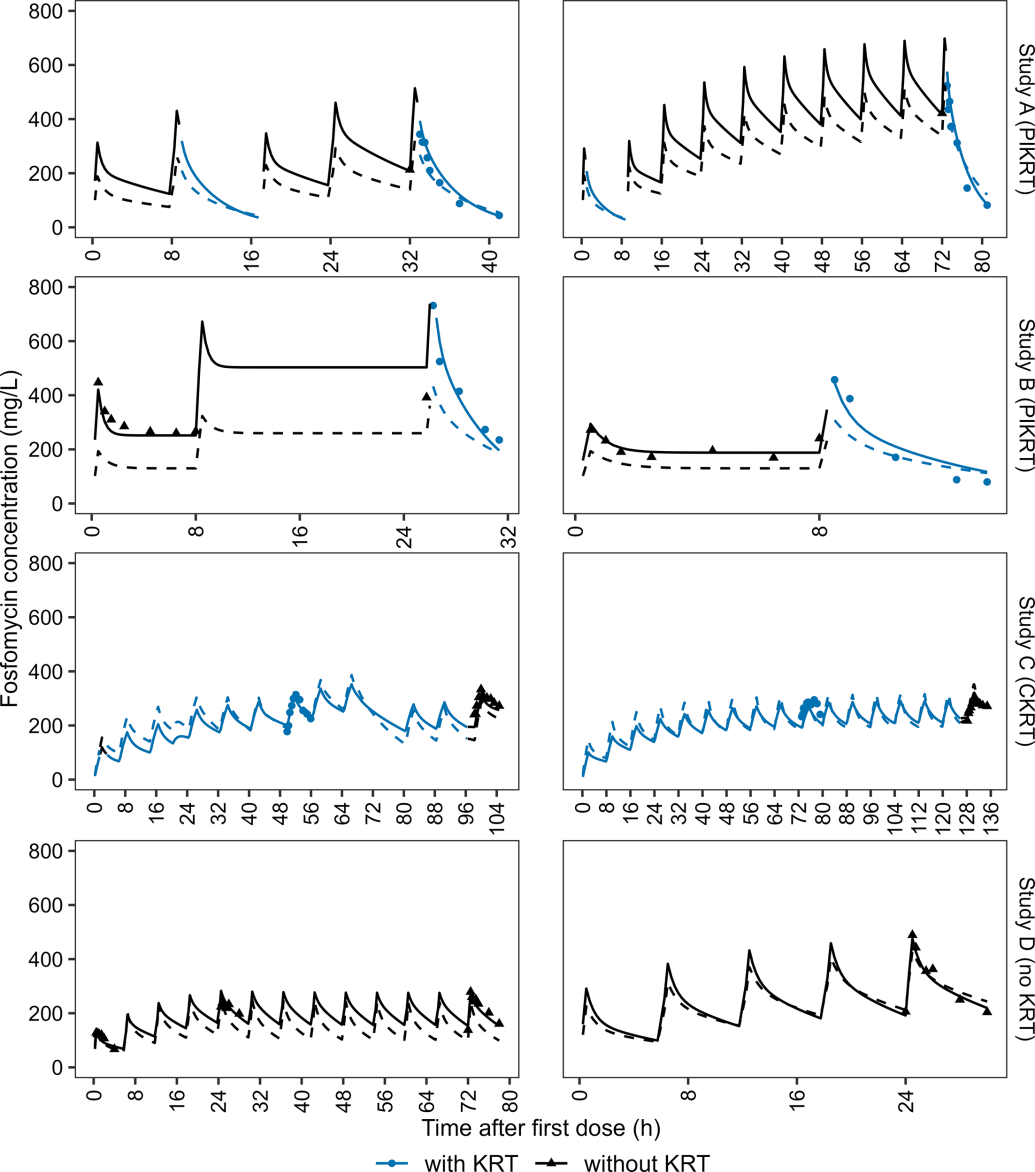
Exemplary fosfomycin concentration–time profiles stratified by KRT and study. Observed and model-predicted data are shown in blue (with KRT) and black (without KRT), respectively. Dots and triangles indicate observations with and without KRT, respectively. Solid and dashed lines represent the respective population and individual predicted fosfomycin concentrations. CKRT, continuous kidney replacement therapy; PIKRT, prolonged-intermittent kidney replacement therapy; KRT, kidney replacement therapy.

The parameters of the final model were estimated precisely (RSEs ≤ 32%, Table 2). To assess the potential influence of study B on final model parameter estimates, we excluded the observed data from study B (reduced data set) for model parameter estimation. The results of this analysis demonstrated the consistency of parameter estimates between the full data set and the reduced data set (Table 2). Goodness-of-fit plots demonstrated adequate descriptive performance of the final model (Fig. S1). Observations and model predictions were randomly scattered around the line of identity, and conditional weighted residuals showed no trend over time. The pcVPC stratified by KRT and study demonstrated good accordance between observed and model-predicted fosfomycin concentrations (Fig. S4 and S5). Additionally, exemplary fosfomycin concentration–time profiles of two randomly selected patients from each study indicated the reasonable descriptive performance of the final model (Fig. 4). Fig. S2 and S3 present fosfomycin concentration–time profiles of all 45 patients.

**TABLE 2:** Parameter estimates of the final model^*a*^^,^^*f*^

	Final model	
	Full data set (*n* = 45)	Reduced data set (*n* = 37)
**Parameter**	Estimate (RSE, %)	Estimate (RSE, %)
Fixed effects		
Body clearance (Θ_CL_), L/h	1.6 (20)^*b*^	1.6 (24)^*b*^
Kidney function (Θ_KF_)	0.869 (25)	0.875 (28)
		
Volume of distribution, L		
Central (Θ_VC_)	23.1 (10)	25.0 (9)
Peripheral (Θ_VP_)	15.4 (18)^*c*^	14.6 (17)^*c*^
Time after the first dose (Θ_T_), min	0.0007 (28)	0.0008 (24)
		
Intercompartmental clearance (Θ_Q_), L/h	12.0 (17)	10.7 (20)
		
Dialysis clearance (Θ_KRT_), L/h	2.0 (15)^*d*^	2.05 (13)^*d*^
Q_D_ (Θ_QD_), mL/min	0.587 (16)	0.488 (19)
		
Random effects: IIV		
Body clearance (η_CL_), %CV	84.6 (14)^*e*^	84.7 (17)^*e*^
Volume of distribution, %CV		
Central (η_VC_)	74.8 (12)^*e*^	81.6 (13)^*e*^
Peripheral (η_VP_)	71.1 (32)^*e*^	51.1 (18)^*e*^
		
Random effects: residual variability		
Proportional, %CV	14.7 (20)	15.0 (21)
Additive, mg/L	21.9 (19)	21.9 (22)

^*a*^ CL_Dialysis_, dialysis clearance; CL_body_, body clearance; CV, coefficient of variation; eGFR_MDRD_, estimated glomerular filtration rate calculated using the Modification of Diet in Renal Disease equation; IIV, interindividual variability; Q, intercompartmental clearance; Q_D_, dialyzate flow rate; TSFD, time since the first dose in minutes; V_P_, peripheral volume of distribution.

^*b*^ $CL_{body}=\theta_{CL}\times (eGFR_{MDRD}/48.4{)}^{\theta_{KF}}\times exp(\eta_{CL})(\times \;0\;if\;study\;B)(\times \;0\;if\;24-h\;urine\;output\;<100\;\;mL).$

^*c*^ $V_{Peripheral}=\theta_{VP}\times (1+TSFD\times \theta_{T})\times exp(\eta_{VP}).$

^*d*^ $CL_{KRT}=\theta_{DIAL}\times (Q_{D}/42{)}^{\theta_{QD}}.$

^*e*^ IIV calculated from$\sqrt{exp(\omega^{2})-1.}\omega =variance.$

^*f*^ η=inter−individualvariation.

### Simulations and optimized dosing recommendations

Figure S6 provides the median predicted fosfomycin concentration–time profiles of 2,000 simulated subjects, stratified by evaluated types of KRT and different dosing regimens, to illustrate fosfomycin exposure, as described by the final model. A dosage of 4 g TID resulted in fosfomycin concentrations exceeding the MIC of 64 mg/L within 24 hours after the first dose for patients without KRT and eGFR_MDRD_ < 120 mL/min/1.73 m^2^ (Fig. S6, left panel). Comparing patients without and with CKRT, CKRT at the treatment start is associated with considerably reduced fosfomycin exposure across the entire dosage range (Fig. S6, middle panel). Moreover, simulations suggest that PIKRT on the second day of treatment effectively prevents critical accumulations of fosfomycin for patients with eGFR_MDRD_ ≤ 60 mL/min/1.73 m^2^ (Fig. S6, left panel).

**Fig 5:**
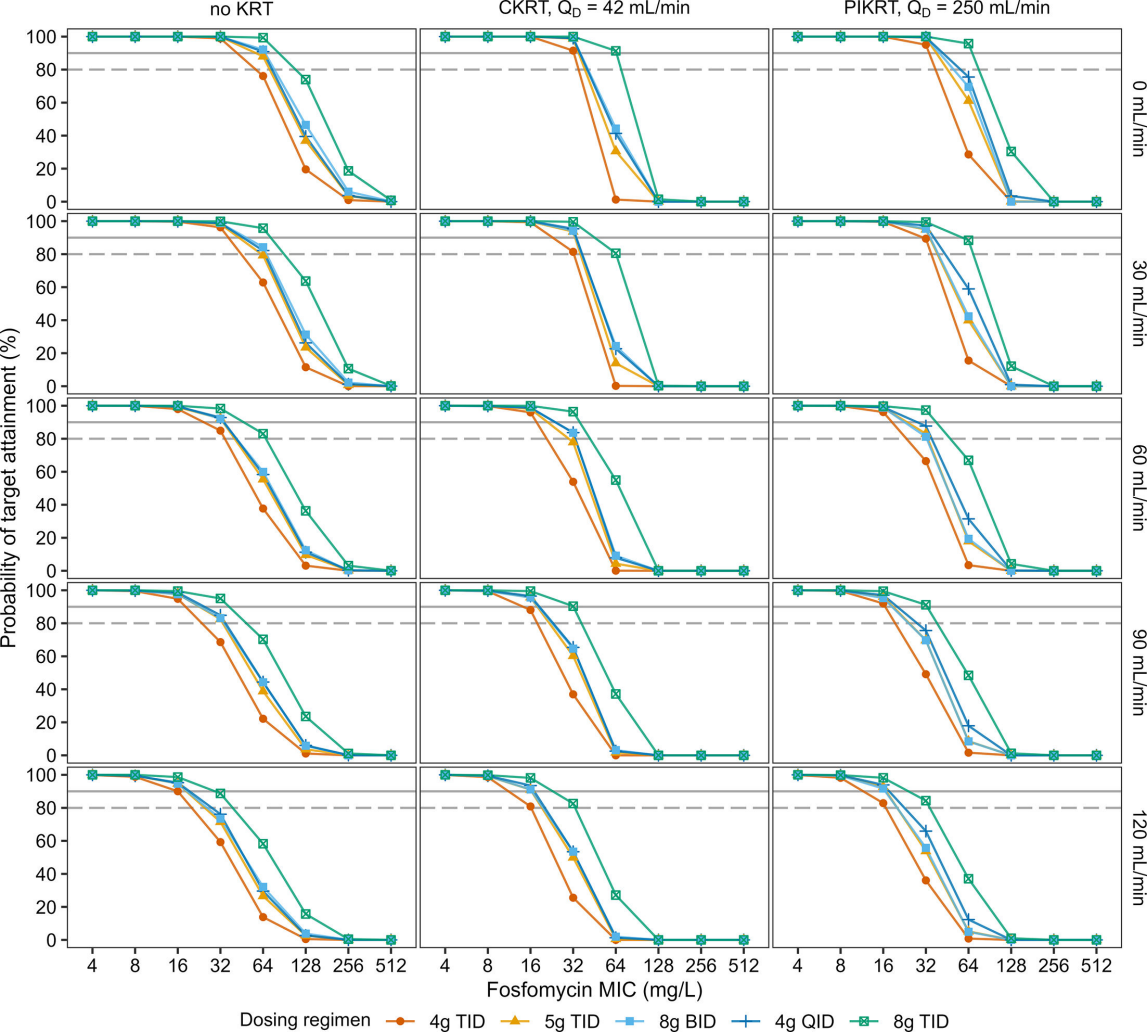
Probability of target attainment for the bactericidal activity (AUC_24-48_/MIC = 83.3) at different MICs, stratified by different types of KRT (columns), varying groups of kidney function (eGFR_MDRD_, rows), and different fosfomycin dosing regimens (colors). The dashed and solid lines indicate PTA targets of 80% and 90%, respectively. AUC_24-48_/MIC, ratio of area under the concentration–time curve from 24–48 hours and MIC; BID, twice daily; CKRT, continuous kidney replacement therapy; eGFR_MDRD_, estimated glomerular filtration rate in mL/min/1.73 m^2^ calculated using the Modification of Diet in Renal Disease equation; MIC, minimum inhibitory concentration; PIKRT, prolonged-intermittent kidney replacement therapy; KRT, kidney replacement therapy; Q_D_, dialyzate flow rate; QID, four times daily; TID, three times daily; PTA, probability of target attainment.

Figure 5 and Fig. S7 show PTA results for bactericidal (AUC_24-48_/MIC ratio = 83.3) and bacteriostatic (AUC_24-48_/MIC ratio = 22.7) activity, respectively. Table 3 provides IV fosfomycin dosing recommendations, as evaluated by the lowest dosage to achieve PTA targets of 80% or 90% (AUC_24-48_/MIC ratio). At the MIC of 32 mg/L, 4 g TID (anuria), 5 g TID (eGFR ≤30 mL/min/1.73 m^2^), and 8 g TID (eGFR ≤90 mL/min/1.73 m^2^) were bactericidal (PTA ≥ 90%) during KRT, whereas 4 g TID (eGFR ≤30 mL/min/1.73 m^2^), 5 g TID (eGFR ≤60 mL/min/1.73 m^2^), and 8 g TID (eGFR ≤90 mL/min/1.73 m^2^) were bactericidal (PTA ≥ 90%) without KRT. Also, 4 g TID was bacteriostatic (PTA ≥ 90%) across all scenarios. At the MIC of 64 mg/L, 8 g TID was bactericidal (PTA ≥ 80%) with KRT (eGFR ≤30 mL/min/1.73 m^2^) and without KRT (eGFR ≤60 mL/min/1.73 m^2^). The bacteriostatic activity (PTA ≥ 90%) was demonstrated for 5 g TID during KRT (eGFR ≤90 mL/min/1.73 m^2^) and without KRT (eGFR ≤120 mL/min/1.73 m^2^).

**TABLE 3:** Dosing recommendations for patients with CKRT (Q_D_ = 42 mL/min) or PIKRT (Q_D_ = 250 mL/min), as well as without KRT, to maintain bactericidal (AUC_24-48_/MIC = 83.3) or bacteriostatic (AUC_24-48_/MIC = 22.7) activity with PTA ≥ 90% (white background) or ≥ 80% (gray background) at three different MICs^*a*^^,*b*^

		MIC = 32 mg/L		MIC = 64 mg/L		MIC = 128 mg/L
	eGFR_MDRD_	Bactericidal	Bacteriostatic	Bactericidal	Bacteriostatic	Bacteriostatic
KRT	(mL/min/1.73 m^2^)	Dosage	Dosage	Dosage	Dosage	Dosage
CKRT or PIKRT	0 (anuria)	4 g TID	4 g TID	8 g TID	4 g TID	5 g TID
CKRT or PIKRT	≤30	5 g TID	4 g TID	8 g TID	4 g TID	5 g TID
CKRT or PIKRT	31–60	8 g TID	4 g TID	N/A^*c*^	4 g TID	8 g TID
CKRT or PIKRT	61–90	8 g TID	4 g TID	N/A	5 g TID	8 g TID
						
No KRT	≤30	4 g TID	4 g TID	8 g TID	4 g TID	4 g TID
No KRT	31–60	5 g TID	4 g TID	8 g TID	4 g TID	5 g TID
No KRT	61–90	8 g TID	4 g TID	N/A	4 g TID	8 g TID
No KRT	91–120	8 g TID	4 g TID	N/A	5 g TID	8 g TID

^*a*^ The recommended dosages represent the lowest dosage that demonstrated PTA ≥ 90% (white background) or ≥ 80% (gray background), stratified by different MICs, treatment with KRT, and varying groups of kidney function.

^*b*^ AUC_24–48_/MIC, ratio of area under the concentration–time curve from 24–48 hours and MIC; CKRT, continuous kidney replacement therapy with Q_D_ = 42 mL/min; eGFR_MDRD_, estimated glomerular filtration rate in mL/min/1.73 m^2^ calculated using the Modification of Diet in Renal Disease equation; MIC, minimum inhibitory concentration; PIKRT, prolonged-intermittent kidney replacement therapy with Q_D_ = 250 mL/min; KRT, kidney replacement therapy; Q_D_, dialyzate flow rate; QID, four times daily; TID, three times daily; PTA, probability of target attainment.

^*c*^ N/A, not applicable.

Figure S8 provides PTA results for the PK/PD index %T_24-48_>MIC. At the MIC of 32 mg/L, 4 g TID (eGFR ≤60 mL/min/1.73 m^2^) and 4 g QID (eGFR ≤120 mL/min/1.73 m^2^) were bactericidal (PTA ≥ 90%) with or without KRT. At the MIC of 64 mg/L, bactericidal activity (PTA ≥ 90%) was demonstrated for 4 g TID (eGFR ≤30 mL/min/1.73 m^2^), 4 g QID (eGFR ≤60 mL/min/1.73 m^2^), and 8 g TID (eGFR ≤90 mL/min/1.73 m^2^) during KRT and 4 g TID (eGFR ≤60 mL/min/1.73 m^2^) or 4 g QID (eGFR ≤90 mL/min/1.73 m^2^) without KRT.

## DISCUSSION

Fosfomycin is a valuable treatment option for serious infections with multidrug-resistant bacteria, but there is limited information guiding dose adaptations for critically ill patients undergoing KRT. The current data set (*n* = 45) covers a wide range of kidney functions, from severely decreased (eGFR_MDRD_ <30 mL/min/1.73 m^2^, *n* = 4) to normal (eGFR_MDRD_ ≥90 mL/min/1.73 m^2^, *n* = 7). Furthermore, for patients receiving KRT, blood samples were available between KRT sessions (study B) or on non-dialysis days (study C), providing favorable conditions for the simultaneous characterization of fosfomycin elimination via body clearance and dialysis clearance.

A two-compartment model described the observed data adequately, in agreement with our former CVVHD model (21) and results from previous PK studies on IV fosfomycin (25, 32–37). Recently, a one-compartment model was proposed for IV fosfomycin in patients with renal impairment, but this study was based on fewer sampling points (38). A higher CL_body_ was estimated in our current work as compared to the CVVHD model (CL_body_ = 1.6 L/h vs 0.26 L/h), which is in line with findings from non-critically ill patients (CL_body_ = 1.42–2.43 L/h) (34, 35) or critically ill patients (CL_body_ = 2.06 L/h) (25). In the CVVHD model, CL_body_ was determined by the measured *Cr*CL. Since this laboratory marker is not frequently measured in routine care, only two of four included studies (C, D) presented measurements of creatinine in urine. The Cockcroft–Gault, MDRD, and CKD-EPI equations estimate the kidney function from serum creatinine level, which was available from three of four included studies (A, C–D).

In this pooled data set, patients undergoing KRT (studies A–C) had a considerably higher body weight than patients not receiving KRT (study D), and hence, the Cockcroft–Gault equation, which incorporates body weight, may overestimate the kidney function in these patients (17, 18). The MDRD and CKD-EPI equations have been shown to overestimate the renal function in critically ill patients (39–43). Potential reasons for this include that serum creatinine is a poor marker for renal function in patients recently admitted to intensive care units (44) and that its production changes with muscle atrophy (41). As the MDRD equation was developed in patients with CKD and the CKD-EPI equation demonstrated better accuracy primarily for eGFR >60 mL/min/1.73 m^2^ (27, 40), we implemented the eGFR_MDRD_ in the final model. Importantly, our cohort presented no statistically significant difference between the median values of eGFR_MDRD_ and eGFR_CKD-EPI_ (*P* > 0.05, data not shown). Nevertheless, to minimize a potential overestimation of the individual kidney function as a result of using a serum creatinine-based equation, CL_body_ was excluded for anuric patients (24 hour urine output <100 mL). Of note, we tested 24-hour urine output, e*Cr*CL_CG,_ and eGFR_CKD-EPI_ for effects on CL_body_, which did not improve the performance of the final model including eGFR_MDRD_.

The Michaels equation (29) is commonly used to describe the effects of Q_D_ and Q_B_ on drug dialysis clearance by estimating drug- and dialyzer-specific mass transfer–area coefficients (K_o_A) at given flow rates. For small molecules like urea and creatinine, K_o_A has been shown to vary with Q_D_ (45–47), which may also apply for fosfomycin. Furthermore, the included studies (A–C) used different dialyzers, each requiring a specific K_o_A, which would have limited the generalizability of our model findings. Therefore, we examined the effects of flow rates using various model structures independent of the Michaelis equation. Identifying Q_D_ as the key variable for CL_KRT_, it is possible to simulate fosfomycin dosing regimens for different forms of KRT by varying Q_D_.

The distribution of fosfomycin varies over time—a finding of the CVVHD model, which was confirmed in the combined model. In the combined model, IIV was supported on V_P_, which has not been included in the CVVHD model. This could explain that the central volume of distribution has been found to increase over time in the CVVHD model, while V_P_ expands over time in the combined model. The observed variation in the fosfomycin distribution is considered physiologically plausible in critically ill patients, due to endothelial dysfunction and capillary leakage (48). In combination with intravenous fluid administration in the intensive care unit, increased interstitial fluid and total body water can be observed in these patients (49). For hydrophilic drugs, such as fosfomycin, this may increase the volume of distribution (50), as previously described for meropenem in critically ill patients (51).

Dimski *et al*. (study A) and Gerecke *et al*. (study B) recommended 5 g fosfomycin TID, with or without a bolus dose of 8 g, for patients receiving PIKRT. Gattringer *et al*. (22) recommended 8 g BID for patients with continuous veno-venous hemofiltration, while our previous work (study C) suggested 5 g TID for patients undergoing CVVHD. In this study, PTA analysis (AUC_24-48_/MIC ratio = 83.3) indicated a benefit for a higher dosage of 8 g TID in patients undergoing CKRT or PIKRT with improved kidney function as 5 g TID was bactericidal at eGFR ≤30 mL/min/1.73 m^2^ and 8 g TID at eGFR ≤90 mL/min/1.73 m^2^ at the MIC of 32 mg/L. In contrast, using the %T_24-48_>MIC, 4 g TID and 8 g TID were found to be bactericidal at the MICs of 32 mg/L and 64 mg/L, respectively. Although PTA decreased at MICs > 64 mg/L using both PK/PD indices, 8 g TID consistently showed bactericidal activity at the MIC of 32 mg/L, which aligns with ECOFF values of key pathogens responsible for bloodstream infections, such as *Escherichia coli* (4 mg/L) and *Staphylococcus aureus* (32 mg/L) (52). Notably, IV fosfomycin is typically used in combination therapy for the treatment of severe infections (14, 15), which can substantially decrease the PK/PD index targets, as was previously demonstrated in an *in vitro* study (53). Moreover, combining fosfomycin and meropenem led to a 16-fold decrease in MIC_90_ (512 mg/L to 32 mg/L) in KPC-2-producing *Klebsiella pneumoniae* isolates compared to fosfomycin monotherapy (54). In critically ill patients, IV fosfomycin dosages up to 24 g/day have been well-tolerated (55). Nevertheless, IV fosfomycin is administered as disodium salt, and electrolyte levels should be regularly monitored during prolonged treatment, particularly in patients with heart failure and high pretreatment sodium levels.

The current work has limitations. A minor part of fosfomycin is excreted with the feces in healthy subjects (56), and this elimination pathway may increase in patients with renal impairment. However, we were not able to include these elimination pathways in the model because data to delineate fosfomycin elimination through urine or feces were not available. Furthermore, only one study (A) comprised post-filter concentration measurements, and another study (B) included single concentration measurements in the dialyzate. Hence, there was limited information supporting a more mechanistic implementation of CL_KRT_ (57). Nevertheless, the availability of fosfomycin concentrations on non-dialysis episodes for patients undergoing KRT enabled the differentiation of fosfomycin elimination through CL_body_ or CL_KRT_. The final model adequately captures the central tendency and variability observed within this pooled data set. However, pcVPC results indicate an overprediction of the observed data at the 50th percentile for study D, likely due to limited and early kidney function measurements. To further characterize fosfomycin PK in critically ill patients, ongoing research should involve frequent, preferably daily, assessments of kidney function and clinical interventions. In this regard, in this study, a time-dependent variation in the fosfomycin distribution was observed. Future studies may further investigate the physiological basis of this finding, considering additional extrinsic or intrinsic factors, such as concomitant fluid administration and daily changes in body weight.

To conclude, this PK analysis contributes to a better understanding of IV fosfomycin elimination in critically ill patients, supporting the optimization of fosfomycin dosing regimens for various patient scenarios with or without different forms of KRT. The covariate analysis on this pooled data set identified eGFR_MDRD_, Q_D,_ and time after the first dose as key determinants for fosfomycin PK in this population, which confirms and extends the findings of our previous work. In patients with severely decreased kidney function during CKRT or PIKRT (eGFR_MDRD_ ≤30 mL/min/1.73 m^2^) and without KRT (eGFR_MDRD_ ≤60 mL/min/1.73 m^2^), 5 g TID may be appropriate for bactericidal activity (MIC = 32 mg/L). In patients with improving kidney function during KRT and without KRT (eGFR_MDRD_ ≤90 mL/min/1.73 m^2^), IV fosfomycin dosing regimens of 8 g TID may be considered for critically ill patients to optimize their treatment against infections with multidrug-resistant bacteria.

## Data Availability

The data set can be made available upon reasonable request from the corresponding author.

## SUPPLEMENTAL MATERIAL

Supplemental material aac.01779-24-s0001.pdf

ASM does not own the copyrights to Supplemental Material that may be linked to, or accessed through, an article. The authors have granted ASM a non-exclusive, world-wide license to publish the Supplemental Material files. Please contact the corresponding author directly for reuse.
