# Supplementary material for: Population pharmacokinetics of intravenous fosfomycin: dose optimization for critically ill patients with and without kidney replacement therapy
Source: Antimicrob Agents Chemother. 2025 May 5;69(6):e01779-24. doi: 10.1128/aac.01779-24 (PMC12135532; doi:10.1128/aac.01779-24)
Supplement: Supplemental material [file aac.01779-24-s0001.pdf]

Population pharmacokinetics of intravenous fosfomycin: Dose optimization for critically ill patients with and without kidney replacement therapy Supplementary Material

Katharina M. Götz,[a,b] Sascha Kreuer,[a,c] Anke-Katrin Volz,[a] Suzanne L. Parker,[d]* Jason A. Roberts,[d,e,f,g] George Dimopoulos,[h] Thomas Dimski,[i] Detlef Kindgen-Milles,[i] Lisa K. V. Beuche,[j] Jan T. Kielstein,[j] Thorsten Lehr[a,b]#

[a] Saarmetrics GmbH, Saarland University, Saarbrücken, Germany

[b] Department of Clinical Pharmacy, Saarland University, Saarbrücken, Germany

[c] Department of Anaesthesiology, Intensive Care and Pain Therapy, Saarland University Medical Center and Saarland University Faculty of Medicine, Homburg, Germany

[d] University of Queensland Centre for Clinical Research, Faculty of Medicine, The University of Queensland, Brisbane, Australia

[e] Herston Infectious Diseases Institute (HeIDI), Metro North Health, Brisbane, Australia

[f] Departments of Pharmacy and Intensive Care Medicine, Royal Brisbane and Women's Hospital, Brisbane, Australia

[g] UR UM 103, University of Montpellier, Division of Anesthesia Critical Care and Emergency and Pain Medicine, Nimes University Hospital, Nimes, France

[h] Third Department of Critical Care Medicine, National and Kapodistrian University of Athens, Medical School, Athens, Greece

[i] Department of Anesthesiology, University Hospital Düsseldorf, Heinrich Heine University Düsseldorf, Düsseldorf, Germany

[j] Medical Clinic V (Nephrology | Rheumatology | Blood Purification), Academic Teaching Hospital Braunschweig, Braunschweig, Germany

#Correspondence to: Thorsten Lehr, Email: thorsten.lehr@uni-saarland.de

Supplementary Materials and Methods

Kidney replacement therapy modalities

In study A,[1] patients were treated with prolonged-intermittent kidney replacement therapy (PIKRT) using the Genius system (Genius, Fresenius Medical Care, Bad Homburg) with a polyamix hemofilter (Polyflux140H, Baxter Deutschland, Unterschleissheim). The patients in study B[2] underwent PIKRT using the GENIUS 90 batch dialysis system (Fresenius Medical care, Bad Homburg, Germany) with a polysulfone low-flux dialyser (Polyflux 17 L Gambro) or a polysulfone high-flux dialyser (FX 60 Fresenius Medical Care, Bad Homburg, Germany; or Polyflux 170 H Gambro, Lund, Sweden). In study C,[3] the patients received continuous veno-venous hemodialysis (CVVHD) using the multiFiltrate Ci-Ca (Fresenius Medical Care, Bad Homburg, Germany) with polysulfone membrane hemofilters (Ultraflux AV 1000S; Fresenius Medical Care).

Table S1. Equations for the calculation of individual kidney function.

**24-h *Cr*CL**			$(UCr \times UVol_{24}) / (SCr \times 1440)$
**e*Cr*CL$_{CG}$**	female		$(140 - age) \times WT \times 0.85 / (72 \times SCr)$
	male		$(140 - age) \times WT / (72 \times SCr)$
**eGFR$_{MDRD}$**	female		$175 \times SCr^{-1.154} \times age^{-0.203} \times 0.742$ (× 1.212 if black)
	male		$175 \times SCr^{-1.154} \times age^{-0.203}$ (× 1.212 if black)
**eGFR$_{CKD\text{-}EPI}$**	female	SCr ≤ 0.7 mg/dL	$144 \times (SCr / 0.7)^{-0.329} \times 0.993^{age}$
	female	SCr > 0.7 mg/dL	$144 \times (SCr / 0.7)^{-1.209} \times 0.993^{age}$
	male	SCr ≤ 0.9 mg/dL	$141 \times (SCr / 0.9)^{-0.411} \times 0.993^{age}$
	male	SCr > 0.9 mg/dL	$141 \times (SCr / 0.9)^{-1.209} \times 0.993^{age}$

Age in years. BSA, body surface are (m^2); 24-h *Cr*CL, urinary creatinine clearance (mL/min/1.73 m^2) calculated using 24-h urine output; e*Cr*CL, estimated creatinine clearance (mL/min) using Cockcroft-Gault equation; eGFR$_{CKD\text{-}EPI}$, estimated glomerular filtration rate (mL/min/1.73 m^2) using CKD-EPI equation; eGFR$_{MDRD}$, estimated glomerular filtration rate (mL/min/1.73 m^2) using MDRD equation; SCr, serum creatinine; UCr, urine creatinine; $UVol_{24}$, 24-h urine output; WT, body weight (kg).

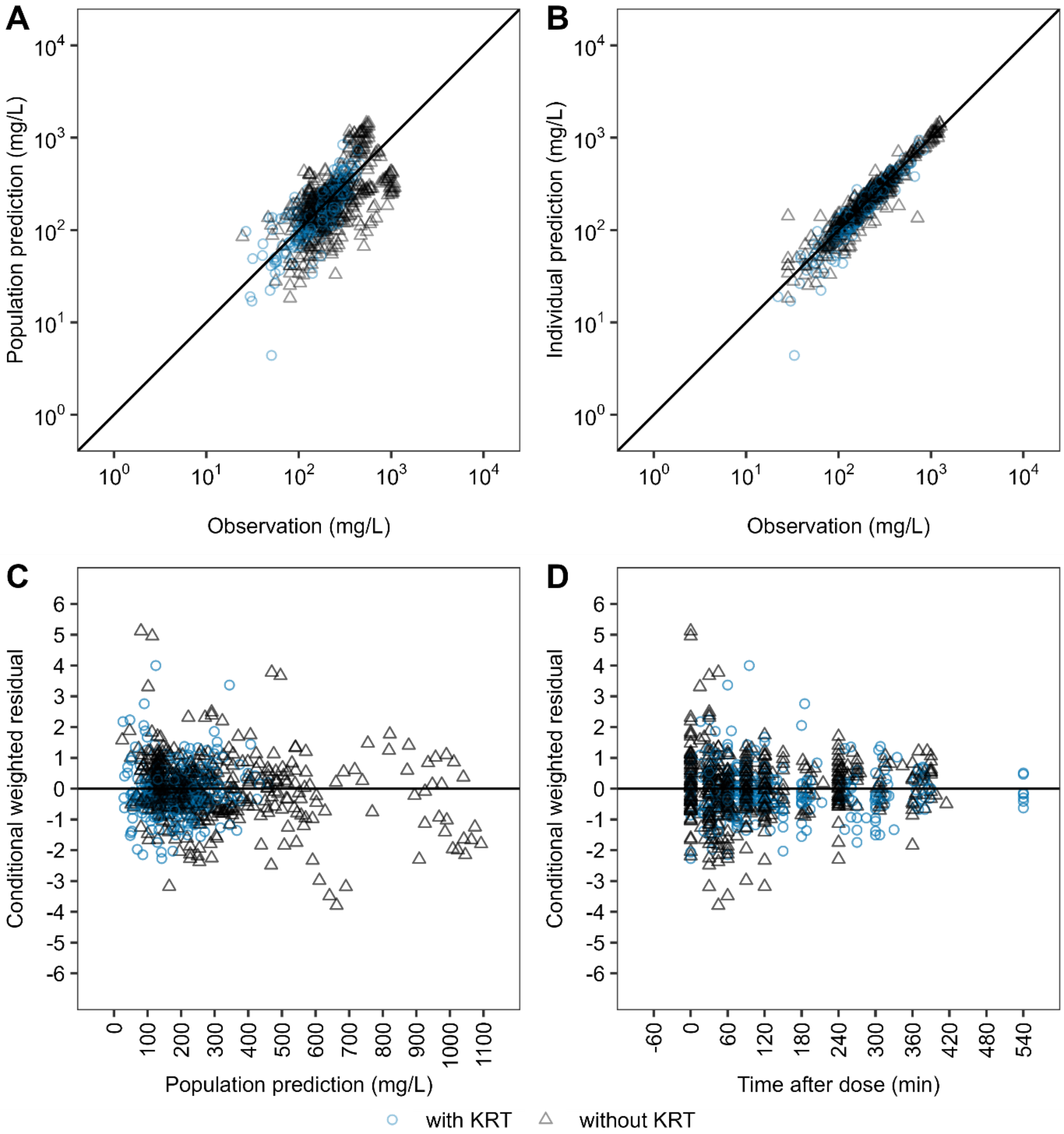

Figure S1. Goodness-of-fit plots for the final model. Observations versus population predictions (**A**) and individual predictions (**B**). Conditional weighted residuals versus population predictions (**C**) and time after dose (**D**). **A, B** The solid lines indicate lines of identity. **A–D** Blue dots and black triangles indicate kidney replacement therapy. KRT, kidney replacement therapy.

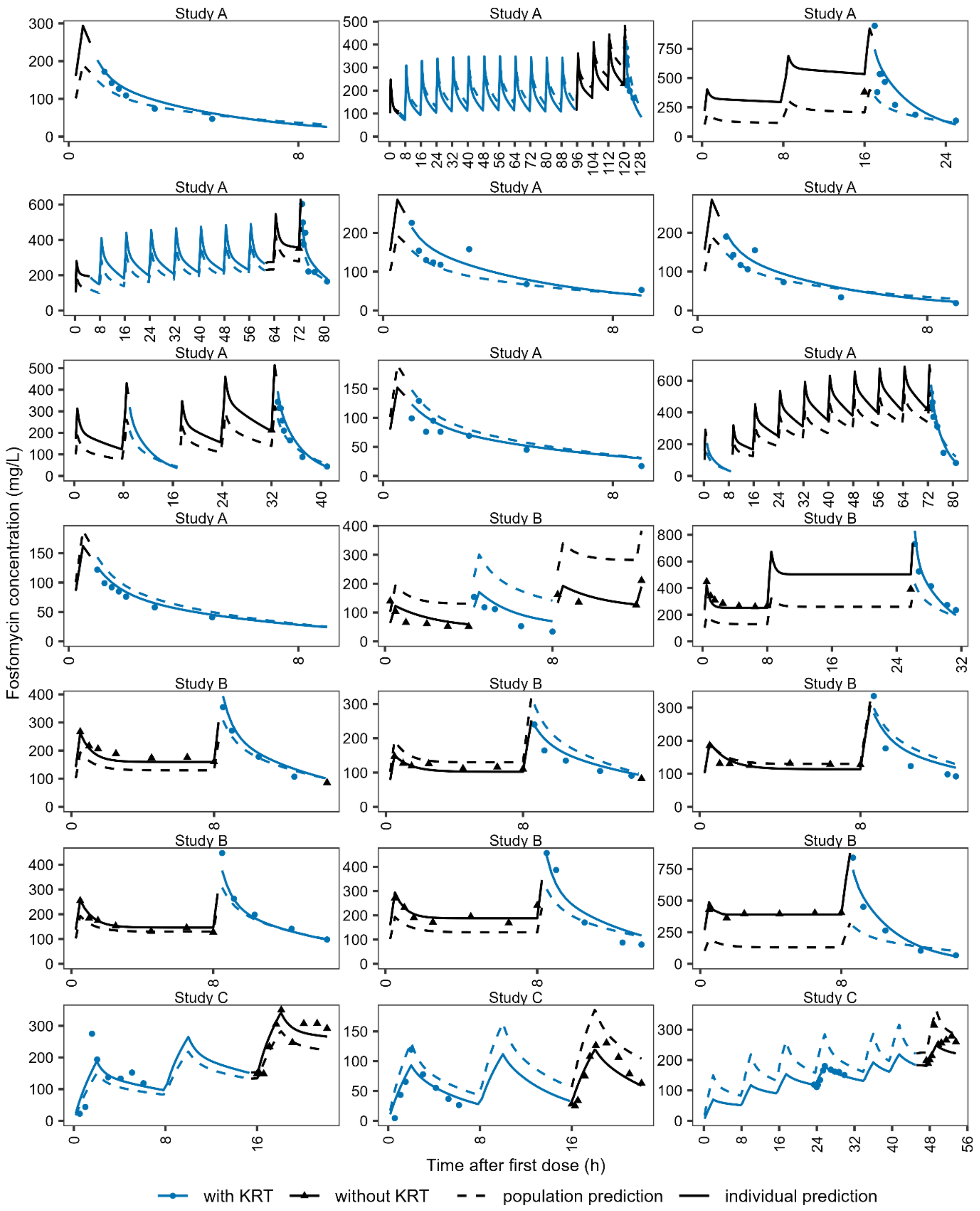

Figure S2. Individual fosfomycin concentration-time profiles for 21 patients. Observed and model predicted data are shown in blue (with KRT) and black (without KRT). Dots and triangles indicate observations. Solid and dashed lines represent the population and individual predicted fosfomycin concentrations. KRT, kidney replacement therapy.

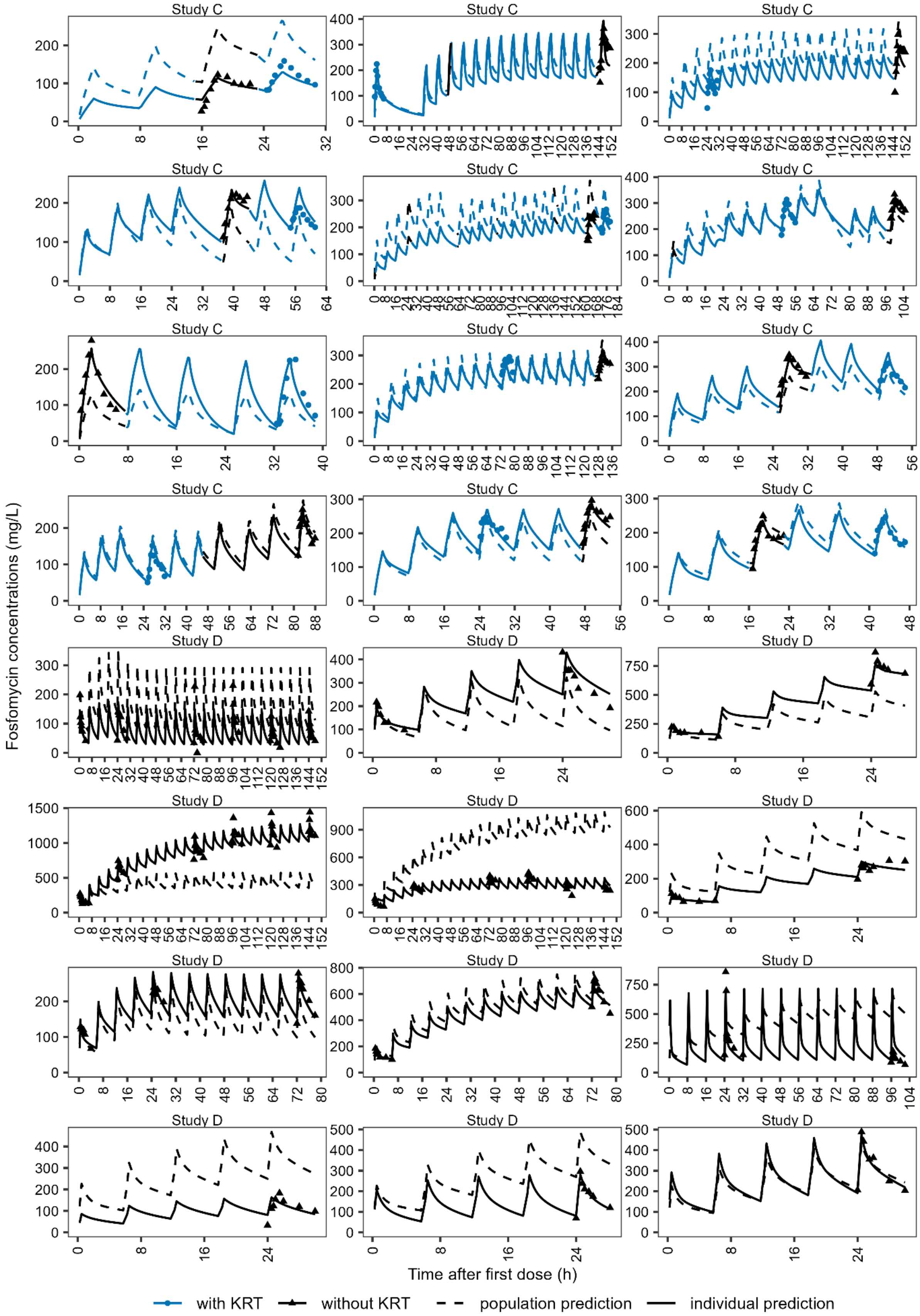

Figure S3. Individual fosfomycin concentration-time profiles for 24 patients. Observed and model predicted data are shown in blue (with KRT) and black (without KRT). Dots and triangles indicate observations. Solid and dashed lines represent the population and individual predicted fosfomycin concentrations. KRT, kidney replacement therapy.

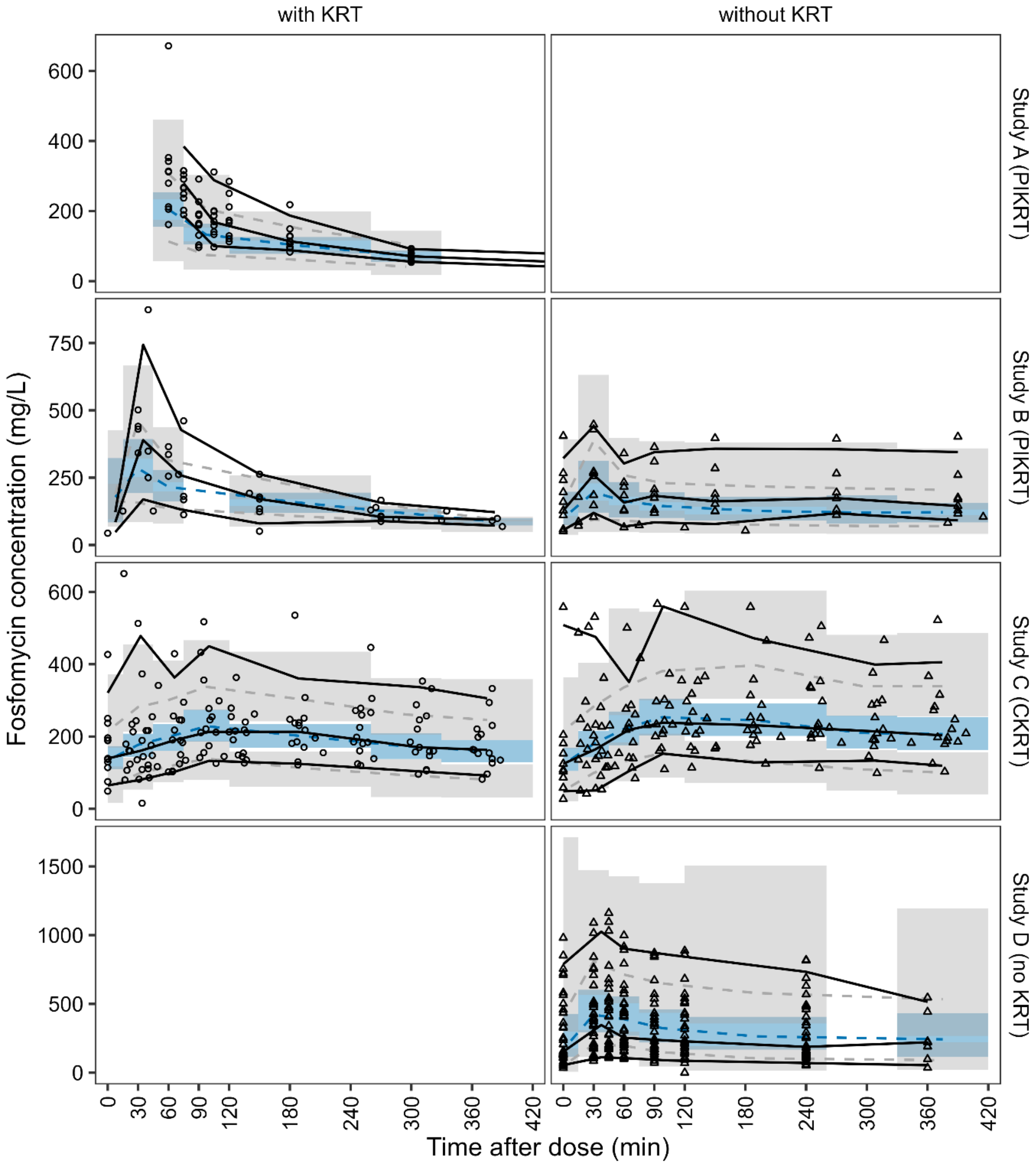

Figure S4. Prediction-corrected visual predictive check for the final model stratified by KRT and study presented on a linear scale. Dots and triangles indicate the prediction-corrected observed concentrations with and without KRT, respectively. Solid lines represent the median, 5th, and 95th percentiles of the prediction corrected observations. The shaded areas show the 95% confidence interval of the median (blue) and the 5th/95th (grey) prediction interval. Dashed lines represent the median, 5th, and 95th percentiles of the prediction corrected simulated data. CKRT, continuous kidney replacement therapy; KRT, kidney replacement therapy; PIKRT, prolonged-intermittent kidney replacement therapy.

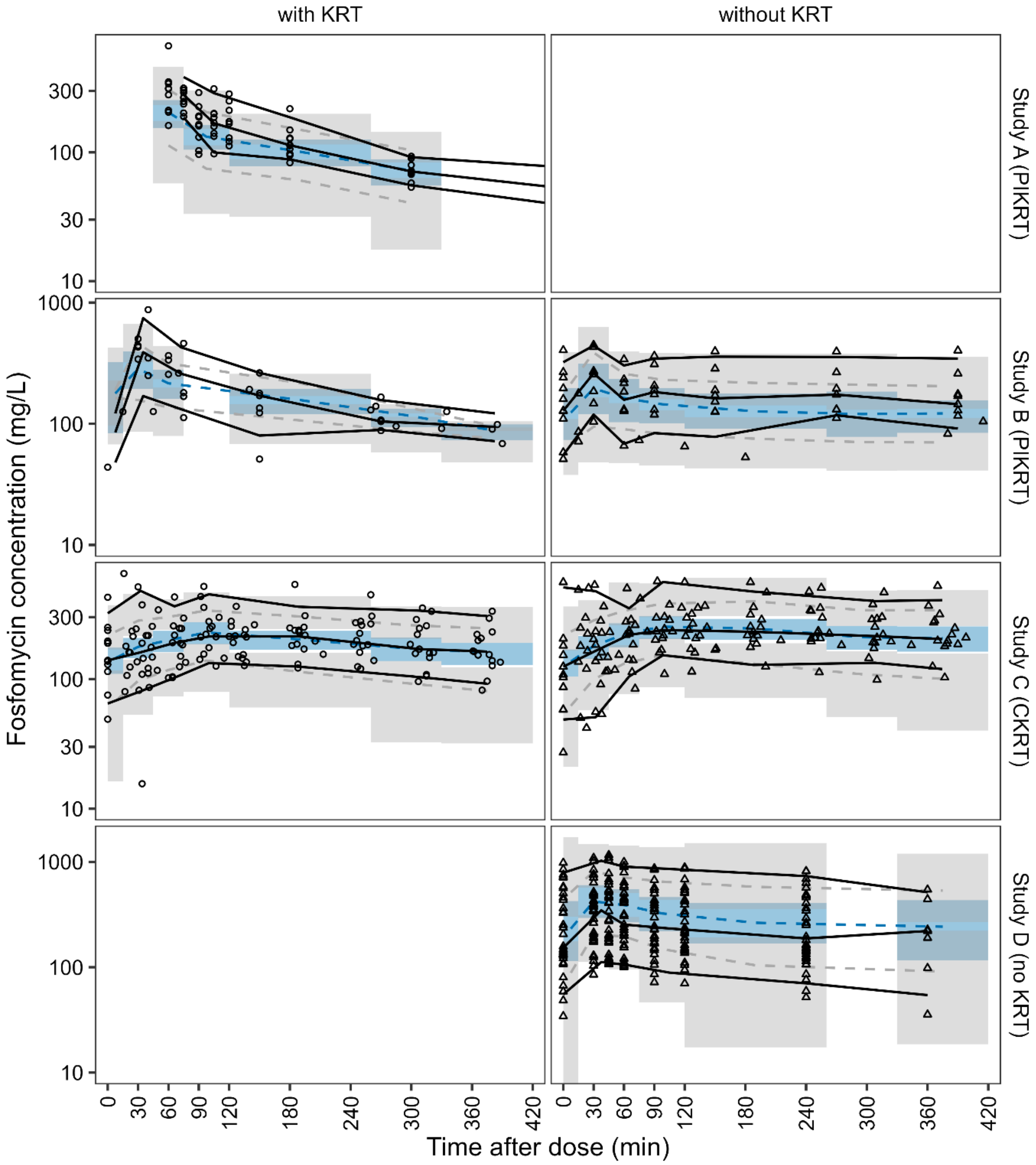

Figure S5. Prediction-corrected visual predictive check for the final model stratified by KRT and study presented on a semi-log scale. Dots and triangles indicate the prediction-corrected observed concentrations with and without KRT, respectively. Solid lines represent the median, 5th, and 95th percentiles of the prediction corrected observations. The shaded areas show the 95% confidence interval of the median (blue) and the 5th/95th (grey) prediction interval. Dashed lines represent the median, 5th, and 95th percentiles of the prediction corrected simulated data. CKRT, continuous kidney replacement therapy; KRT, kidney replacement therapy; PIKRT, prolonged-intermittent kidney replacement therapy.

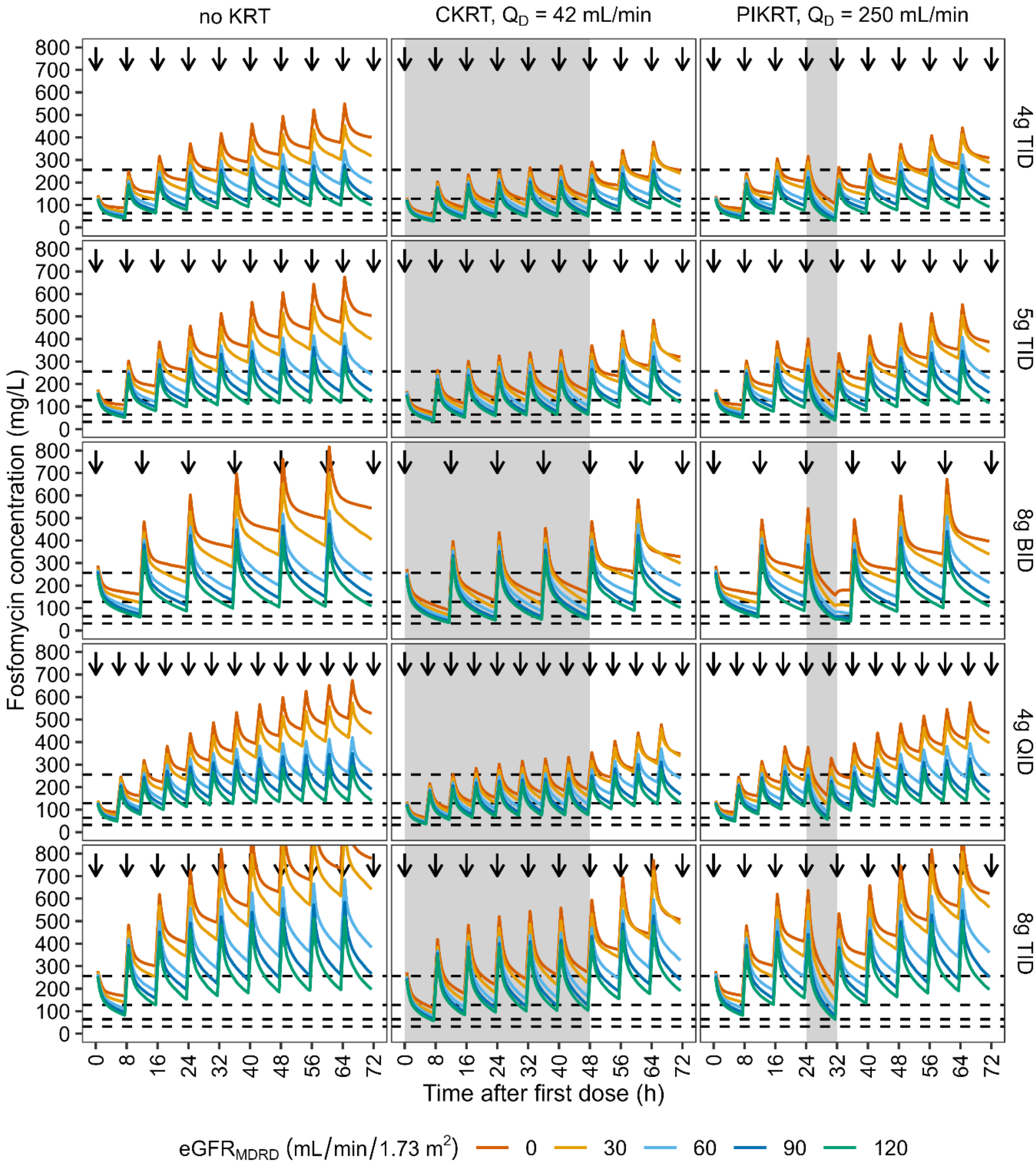

Figure S6. Simulations of different fosfomycin dosing regimens (rows), different types of KRT (columns), and varying groups of kidney function (colors). The solid lines represent the median of n = 2000 simulated fosfomycin concentrations. The grey shaded areas depict periods of KRT. The black arrows indicate intravenous dosages of fosfomycin. In each scenario, the first dose was an intravenous bolus injection, whereas all other doses were 30 min infusions. The dashed lines indicate MICs of 32, 64, 128, and 256 mg/L. BID, twice daily; CKRT, continuous kidney replacement therapy; eGFR$_{MDRD}$, estimated glomerular filtration rate calculated using Modification of Diet in Renal Disease equation; KRT, kidney replacement therapy; MIC, minimal inhibitory concentrations; PIKRT, intermittent kidney replacement therapy; Q_D, dialysate flow rate; QID, four times daily; TID, three times daily.

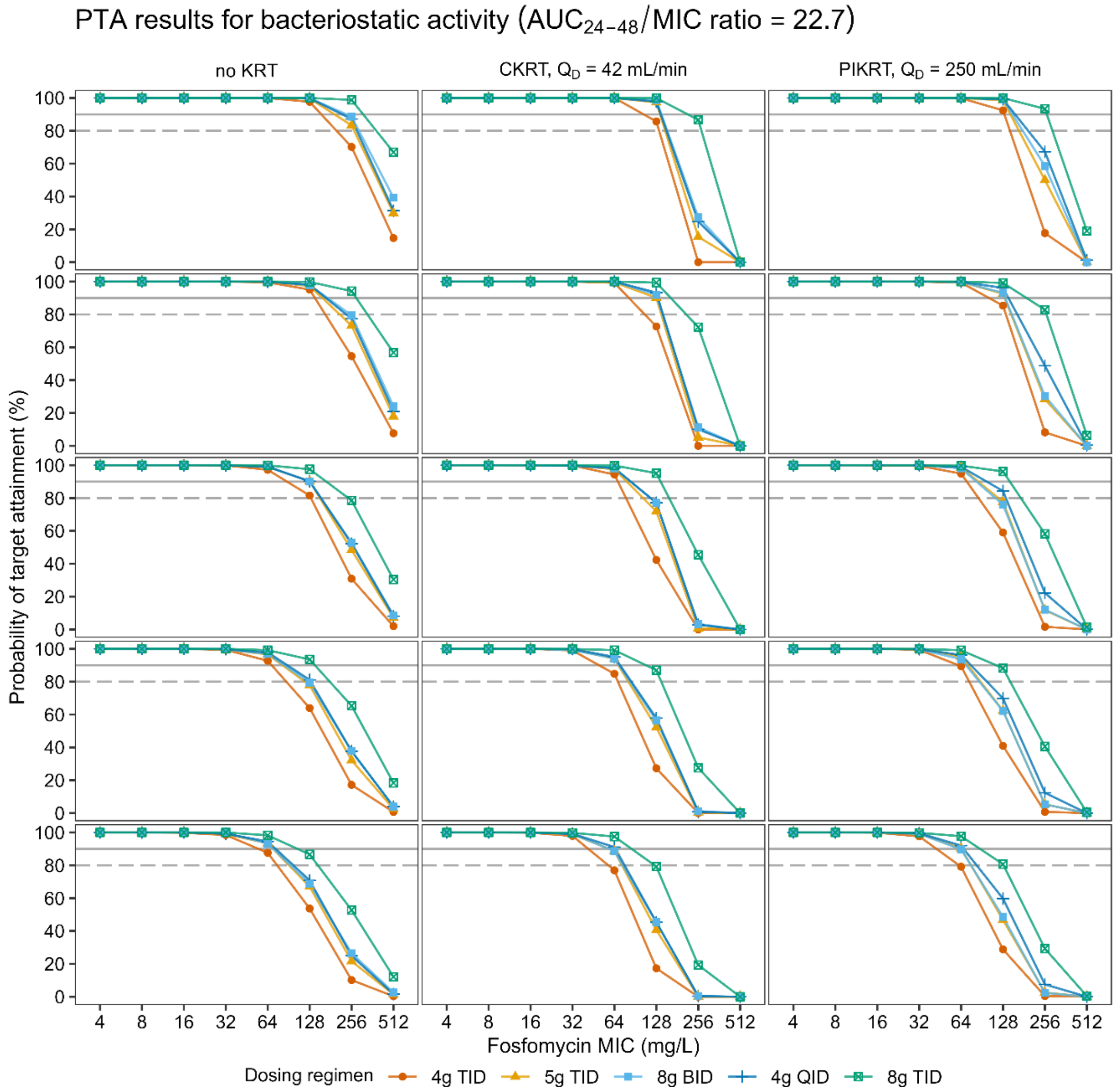

Figure S7. Probability of target attainment for bacteriostatic activity ($AUC_{24\text{-}48}$/MIC = 22.7) at different MICs, stratified by different types of KRT (columns), varying groups of kidney function ($eGFR_{MDRD}$, rows) and different fosfomycin dosing regimens (colors). The dashed and solid lines indicate PTA targets of 80% and 90%. $AUC_{24\text{-}48}$/MIC, ratio of area under the concentration-time curve from 24–48 h and MIC; BID, twice daily; CKRT, continuous kidney replacement therapy; $eGFR_{MDRD}$, estimated glomerular filtration rate in mL/min/1.73 m^2 calculated using Modification of Diet in Renal Disease equation; MIC, minimum inhibitory concentration; PIKRT, prolonged-intermittent kidney replacement therapy; KRT, kidney replacement therapy; Q_D, dialysate flow rate; QID, four times daily; TID, three times daily; PTA, probability of target attainment.

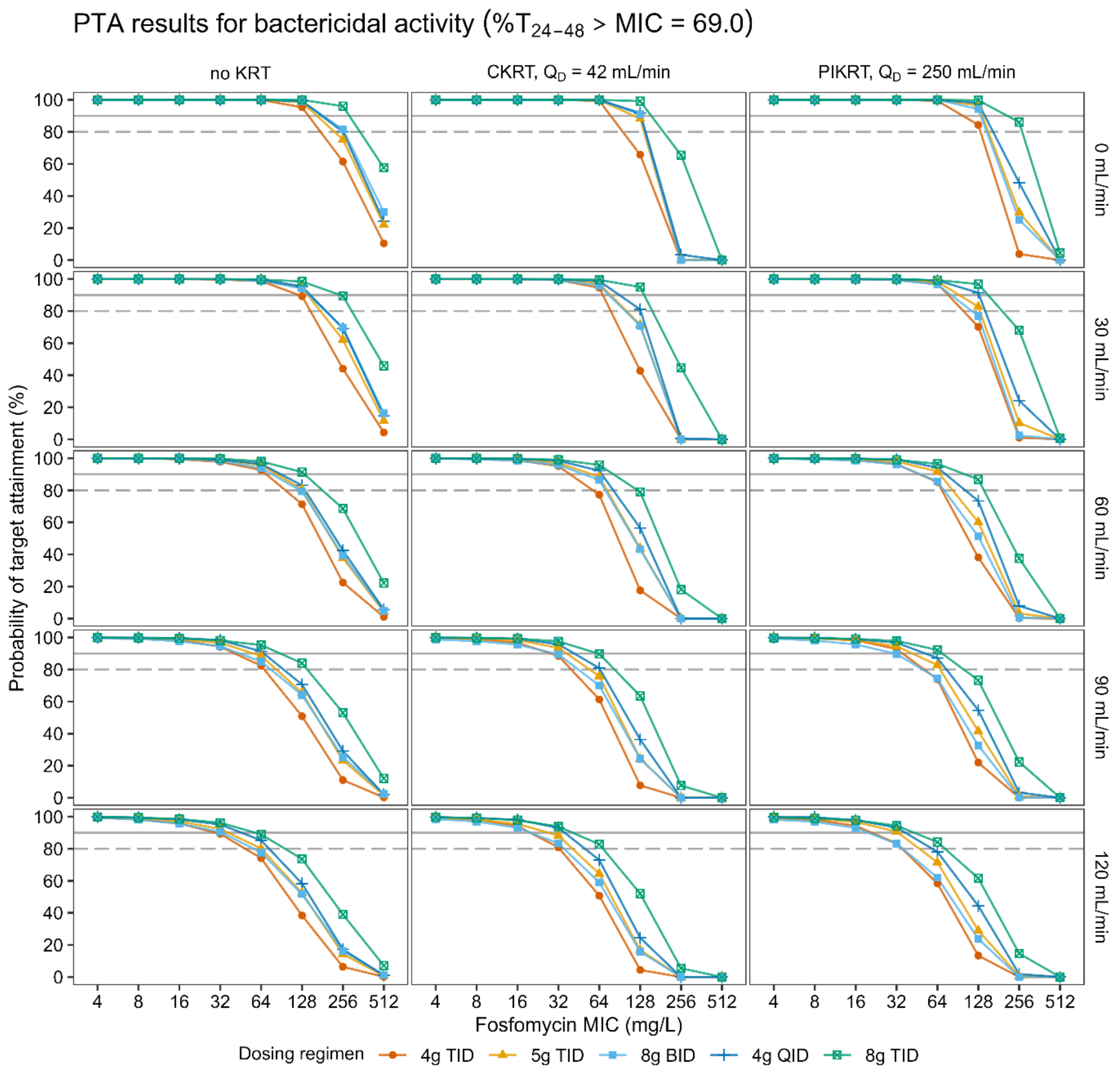

Figure S8. Probability of target attainment for bactericidal activity (%T>MIC = 69.0) at different MICs, stratified by different types of KRT (columns), varying groups of kidney function ($eGFR_{MDRD}$, rows) and different fosfomycin dosing regimens (colors). The dashed and solid lines indicate PTA targets of 80% and 90%. BID, twice daily; CKRT, continuous kidney replacement therapy; $eGFR_{MDRD}$, estimated glomerular filtration rate in mL/min/1.73 m^2 calculated using Modification of Diet in Renal Disease equation; MIC, minimum inhibitory concentration; PIKRT, prolonged-intermittent kidney replacement therapy; KRT, kidney replacement therapy; Q_D, dialysate flow rate; QID, four times daily; TID, three times daily; $\%T_{24-48}$, percentage of time that fosfomycin concentrations exceeded the MIC between 24–48 h; PTA, probability of target attainment.

Supplementary References

1. Dimski T, Brandenburger T, Janczyk M, Slowinski T, MacKenzie C, Kindgen-Milles D. Elimination of fosfomycin during dialysis with the Genius system in septic patients. *Sci Rep*. 2021;11(1):1-6. doi:10.1038/s41598-021-91423-9
2. Gerecke LKV, Schmidt JJ, Hafer C, et al. Fosfomycin single- and multiple-dose pharmacokinetics in patients undergoing prolonged intermittent renal replacement therapy. *J Antimicrob Chemother*. 2021;77(1):169-173. doi:10.1093/jac/dkab357
3. Hüppe T, Götz KM, Meiser A, et al. Population pharmacokinetic modeling of multiple-dose intravenous fosfomycin in critically ill patients during continuous venovenous hemodialysis. *Sci Rep*. 2023;13(1):1-13. doi:10.1038/s41598-023-45084-5
4. Parker SL, Frantzeskaki F, Wallis SC, et al. Population pharmacokinetics of fosfomycin in critically ill patients. *Antimicrob Agents Chemother*. 2015;59(10):6471-6476. doi:10.1128/AAC.01321-15
5. Lepak AJ, Zhao M, Vanscoy B, et al. In Vivo pharmacokinetics and pharmacodynamics of ZTI-01 (fosfomycin for injection) in the neutropenic murine thigh infection model against Escherichia coli, klebsiella pneumoniae, and pseudomonas aeruginosa. *Antimicrob Agents Chemother*. 2017;61(6):1-11. doi:10.1128/AAC.00476-17
